# Optimizing percutaneous vertebroplasty: extra-facet puncture for osteoporotic vertebral compression fractures

**DOI:** 10.1186/s13018-023-04368-2

**Published:** 2023-11-22

**Authors:** Huo-Liang Zheng, Bo Li, Qin-Yu Jiang, Lei-Sheng Jiang, Xin-Feng Zheng, Sheng-Dan Jiang

**Affiliations:** 1 Department of Clinic of Spine Center, Xinhua Hospital, Shanghai Jiaotong University School of Medicine, Shanghai, 200092 China; 2 Shanghai Weiyu High School, Shanghai, 200231 China

**Keywords:** Extra-facet puncture, Percutaneous vertebroplasty, Facet joint violation, Bone cement distribution, Residual back pain

## Abstract

**Purpose:**

To assess the safety and efficacy of the extra-facet puncture technique applied in unilateral percutaneous vertebroplasty (PVP) for treating osteoporotic vertebral compression fractures.

**Methods:**

Demographics (age, gender, body mass index and underlying diseases) were recorded for analyzing. Visual analog scale (VAS) and Oswestry Disability Index (ODI) scores as well as their corresponding minimal clinically important difference (MCID) were used to evaluate clinical outcomes. The segmental kyphotic angle, the vertebral compression ratio and bone cement distribution pattern were evaluated by the plain radiographs. The facet joint violation (FJV) was defined by the postoperative computed tomography scan. Binary logistic regression analysis was performed to investigate relationships between multiple risk factors and residual back pain.

**Results:**

VAS and ODI scores in both traditional puncture group and extra-facet puncture group were significantly decreased after PVP surgery (*p* < 0.05). However, no significant difference was observed between the two groups according to VAS and ODI scores. The proportion of patients achieving MCID of VAS and ODI scores was higher in extra-facet puncture group as compared to traditional puncture group within a month (*p* < 0.05). Finally, multivariate logistic regression analysis showed that FJV (odds ratio 16.38, *p* < 0.001) and unilateral bone cement distribution (OR 5.576, *p* = 0.020) were significant predictors of residual back pain after PVP surgery.

**Conclusions:**

Extra-facet puncture percutaneous vertebroplasty can decrease the risk of FJV and it also has the advantage of more satisfied bone cement distribution.

## Introduction

As the progressive aging of the general population, osteoporosis characterized by reduced bone mass and persistent increased fracture risk has become a growing socioeconomic and medical issue that requires proactive treatment [1–6]. Osteoporotic vertebral compression fracture (OVCF) is one of the most common osteoporotic fractures [7, 8]. In particular, OVCFs are alarmingly prevalent in patients over 50 years old [9]. Percutaneous vertebroplasty (PVP), a minimally invasive procedure, is widely used for treating OVCF in the elderly [10].

Despite about 87% patients had pain relief as reported in a systematic review, residual pain still remained in minority but significant proportion of patients underwent PVP surgery [11]. Studies targeting the causes of residual back pain following PVP surgery have identified several risk factors such as low bone density, low bone cement volume, uneven bone cement distribution, intravertebral vacuum cleft and posterior fascia oedema et al. [12–16]. Recently, facet joint violation has attracted attention during PVP surgery [17]. As yet, how to avoid facet joint violation during the surgical procedure has been little investigated.

We proposed an extra-facet puncture for PVP to avoid facet joint violation. The purpose of this study is to demonstrate the safety and efficacy of the extra-facet puncture for PVP in the treatment of OVCFs.

## Methods

### Patients

This study was a prospective analysis of patients underwent PVP surgery in Jan 2018–Nov 2020. Surgical procedure was carried out for acute OVCFs as determined by magnetic resonance imaging (MRI). Our study employed a prospective design, and patients were allocated to groups using a randomization method. We employed a computer-generated randomization scheme to ensure the random assignment of patients into the traditional puncture and extra-facet puncture groups. This randomization process was conducted by an independent researcher who was not directly involved in patient recruitment or data collection. The inclusion criteria were as follows: (1) Thoracolumbar vertebral compression fractures below T3 without other fractures; (2) Patients had an onset of back pain with minor trauma or no trauma history; (3) Males aged ≥ 55 or females who were postmenopausal; (4) Preoperative radiological examinations confirmed fresh OVCFs showing high signal in T2-weighted fat-suppressed MRI images; (5) Bone mineral density of the lumbar spine was measured by dual-energy X-ray absorptiometry (DXA), and *T* value < − 2.5 standard deviation (SD). The exclusion criteria were (1) Thoracolumbar infections, thoracolumbar neoplastic fractures, and severe trauma occurring before enrollment; (2) Known malignancies such as multiple myeloma et al. This study was approved by the institutional ethical committee of our hospital and was performed according to the principles of the Declaration of Helsinki of 1975 (Clinical trial number: XHEC-F-2016-189).

### Procedures

All PVP were performed by an experienced surgeon (Sheng-Dan Jiang). The patient was prone on the operating table. After sterilizing the surgical incision site three times and covering with surgical drapes, the entry point was determined according to the preoperative grouping. In traditional puncture group, the entry point was selected in the lateral margin of the pedicles at 10 o’clock on the left side or 2 o’clock on the right side. In extra-facet puncture group, the entry point should locate at the lateral region of facet joint, and the distance from the enter point to the lateral margin of the pedicles was determined preoperatively according to the CT scan. Under fluoroscopy monitoring by C-arm, the puncture needle was placed in the fractured vertebral body. Following that, polymethylmethacrylate (PMMA) was carefully injected into the fractured vertebra under fluoroscopic guidance. The analgesic was only administered orally on the day of surgery.

### Imaging study

**Fig. 1:**
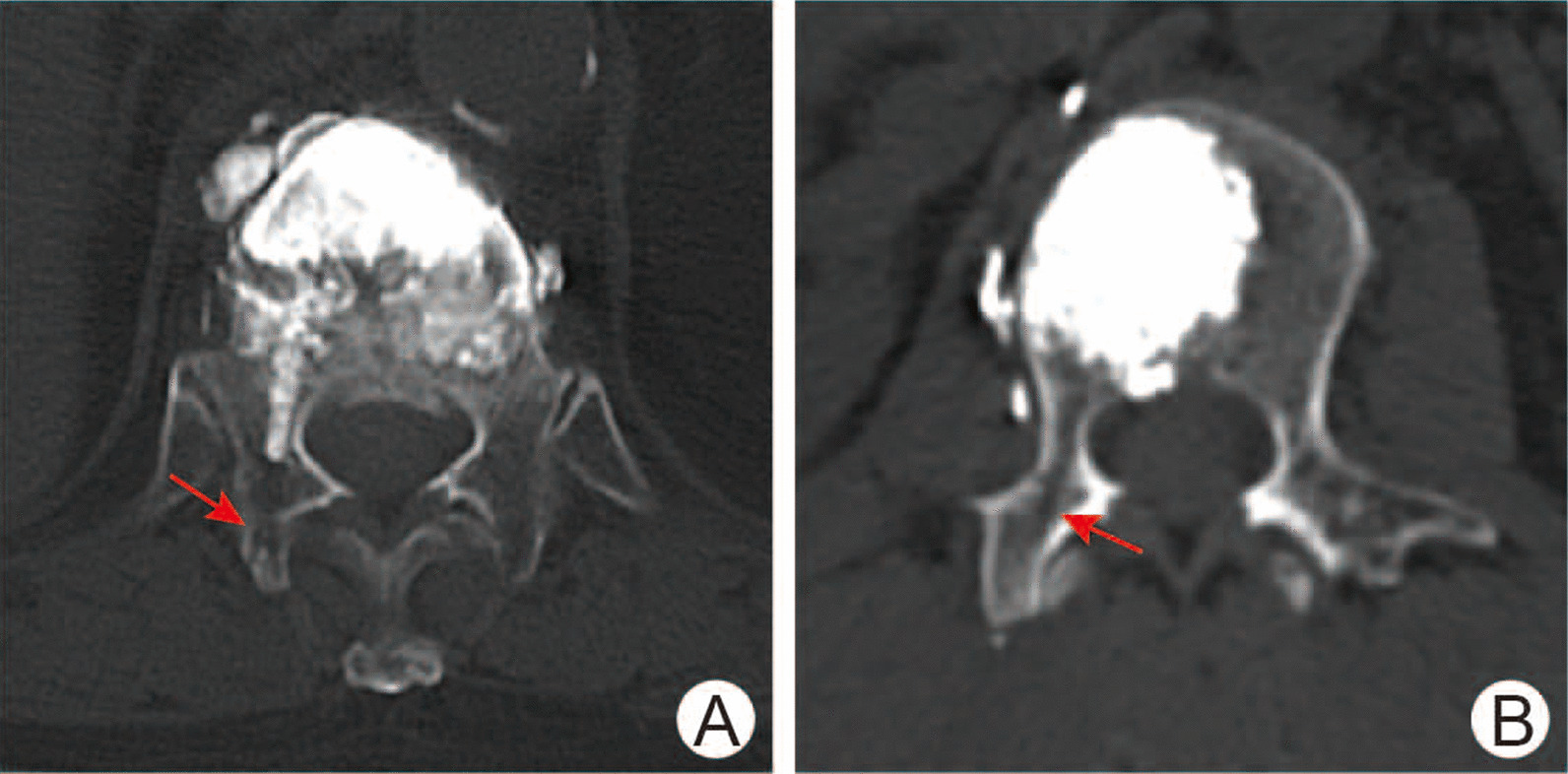
CT transversal view of the trajectory after PVP surgery. **A** Male, 58 years old, who suffered a T12 vertebral fracture and accepted traditional puncture PVP surgery. **B** Male, 81 years old, who suffered a L2 vertebral fracture and accepted extra-facet puncture PVP surgery

Preoperatively, 1 day after surgery, 3 months after surgery and 1 year after surgery, X-ray were taken to measure the vertebral heights, segmental kyphotic angle and the distribution of bone cement. The segmental kyphotic angle is defined as the angle between the superior endplate of the vertebra one level above the treated vertebra and the inferior endplate of the vertebral body one level below the treated vertebra [18]. Bone cement distribution is classified the symmetrical distribution, the eccentric distribution and the unilateral distribution. The symmetrical distribution is defined as bone cement distributing in both sides of the vertebral body and exhibiting bilateral symmetry. The eccentric distribution is defined as bone cement distributing in both sides of the vertebral body but exhibiting predominant unilateral distribution. Bone cement distributing in one side of the vertebral body is considered as the unilateral distribution. Also, the vertebral compression ratio was calculated according to the adjacent anterior vertebral heights [19]. Preoperative CT and MRI scans were carried out to provide essential evidence for clinical decision-making. Moreover, the postoperative CT scan was performed to define whether the facet joint was violated during PVP procedure (Fig. 1).

### Clinical outcomes

The visual analog scales (VAS) were used to assess the intensity of back pain prior to surgery, 1 day, 1 week, 1 month, 3 months and 1 year after surgery. Also, Oswestry Disability Index (ODI) questionnaire was also completed at the same time for evaluating the low back pain. Moreover, the closest minimally clinical important difference (MCID) was used for evaluating the efficacy of surgical treatment—10 for the ODI and 3 for VAS of back [20].

### Agreement phase

A third observer objectively recorded all relevant data assessed by two independent observers (Huo-Liang Zheng and Shao-Kuan Song). The weighted kappa values for the intra- and interobserver agreements were 0.95 and 0.91, respectively. Consensus was reached after discussion for disagreement.

### Statistical analysis

Data were analyzed with IBM SPSS Statistics 26. The continuous variables are presented as mean ± SD. Checking for normality was conducted by the Shapiro–Wilk test. The Mann–Whitney *U* test was used for nonparametric variables compared between two groups, whereas the t test was used for parametric variables. Besides that, the chi-square test and the Fisher’s exact test was used for analyses of distribution. Logistic regressions with multivariable were used to calculate odds ratios (OR). *p* values < 0.05 were considered significant.

## Results

### General characteristics of the patients

In total, 386 patients met the inclusion criteria while 101 refused further participation in the study. As a result, 285 patients were enrolled in this study, with 151 in traditional puncture procedure and 134 in extra-facet puncture procedure. Within one year of surgery, 13 out of 285 patients suffered a second fracture (7 in traditional puncture procedures and 6 in extra-facet puncture procedures). In addition, 1 patient underwent pedicle screw fixation after experiencing nonunion in the extra-facet puncture group. At last, one-year follow-up data were available for 112 patients in traditional puncture group and 105 patients in extra-facet puncture group. In traditional puncture group and extra-facet puncture group, the prevalence of comorbidities was not significantly different (*p* < 0.05). Hypertension is one of the most common comorbidities which occupies a large fraction of patients both in the traditional puncture group (48, 42.8%) and extra-facet puncture group (46, 43.8%). Other baseline characteristics were also similar between the two groups (Table 1).

**Table 1:** Descriptive statistics of the subjects in the study (*x* ± *s*, *n* = 217)

Characteristics	t-PVP (*n* = 112)	ef-PVP (*n* = 105)	*p*
Age at surgery	72.48 ± 6.68	72.15 ± 7.26	0.92^U^
Gender			
Female	92 (82.14%)	80 (75.47%)	0.32^F^
Male	20 (17.86%)	25 (23.81%)	
BMI (kg/m^2^)	24.36 ± 4.31	24.17 ± 4.05	0.74^T^
Hypertension	48 (42.86%)	46 (43.81%)	0.89^F^
Diabetes	14 (12.50%)	8 (7.62%)	0.27^F^
CHD	8 (7.14%)	7 (6.67%)	> 0.99^F^
BMD (*T*-score)	− 3.18 ± 0.45	− 3.24 ± 0.39	0.22^U^
No. of fractures			
One	104 (92.86%)	92 (87.62%)	0.11^C^
Two	5 (4.46%)	12 (11.43%)	
Three	3 (2.68%)	1 (0.95%)	
Fracture site	123	119	
T9	3 (2.43%)	1 (0.84%)	0.83^C^
T10	7 (5.69%)	7 (5.88%)	
T11	8 (6.50%)	14 (11.76%)	
T12	30 (24.39%)	30 (25.21%)	
L1	37 (30.08%)	34 (28.57%)	
L2	24 (19.51%)	17 (14.29)	
L3	7 (5.69%)	7 (5.88%)	
L4	6 (4.88%)	7 (5.88%)	
L5	1 (0.81%)	2 (1.68%)	
Follow-up time (months)	14.66 ± 1.88	14.93 ± 2.02	0.33^U^

The symbol ‘U’ indicates the data was analyzed by the unpaired Mann–Whitney U test; The symbol ‘T’ indicates the data was analyzed by the unpaired t test; The symbol ‘F’ indicates the data was analyzed by the Fisher’s exact test; The symbol ‘C’ indicates the data was analyzed by the Chi-square test

*t-PVP* Traditional puncture percutaneous vertebroplasty, *ef-PVP* Extra-facet puncture percutaneous vertebroplasty, *BMI* Body Mass Index, *CHD* Coronary Artery Atherosclerotic Heart Disease

Statistical analysis revealed no difference between the two groups of patients according to their age, gender, bone density and BMI (*p* > 0.05). There was single-segment PVP in 196 patients, two-segment PVP in 17 patients, and three-segment PVP in 4 patients. Most common level of surgery was L1 (N = 71, 29.3%) followed by T12 (N = 60, 24.8%) and L2 (N = 41, 16.9%) (Table 1). The average volume of injected cement per vertebral body was 6.08 ± 1.44 mL in traditional puncture group and 5.95 ± 1.51 mL in extra-facet puncture group (P = 0.46). Cement leakage was seen in 87 (70.73%) out of the 123 treated vertebrae in traditional puncture group while 84 (70.59%) out of the 119 in extra-facet puncture group. On average, it took 2.4 ± 1.1 days from diagnosis to surgery (range, 1–7 days). Preoperative VAS, ODI, posterior fascia oedema ratio, intravertebral vacuum cleft ration, segmental kyphotic angle and vertebral compression ratio was essentially identical between the two groups (Table 2, *p* > 0.05).

**Table 2:** Comparisons of preoperative parameters between traditional puncture PVP and extra-facet puncture PVP(x ± s)

Parameter	t-PVP	ef-PVP	*p*
No. of patients	112	105	
VAS	7.58 ± 1.21	7.59 ± 1.17	0.95^U^
ODI	63.66 ± 10.71	61.93 ± 11.30	0.25^T^
Posterior fascia oedema	6 (5.36%)	6 (5.71%)	> 0.99^F^
No. of vertebrae	123	119	
Segmental kyphotic angle	14.55 ± 4.87	14.83 ± 5.33	0.67^T^
Intravertebral vacuum cleft	10 (8.93%)	7 (6.67%)	0.62^F^
Vertebral compression ratio	30.32 ± 11.44	31.62 ± 11.48	0.41^U^

The symbol ‘U’ indicates the data was analyzed by the unpaired Mann–Whitney U test; The symbol ‘T’ indicates the data was analyzed by the unpaired t test; The symbol ‘F’ indicates the data was analyzed by the Fisher’s exact test

*t-PVP* Traditional puncture percutaneous vertebroplasty, *ef-PVP* Extra-facet puncture percutaneous vertebroplasty, *VAS* Visual analog scale, *ODI* The Oswestry Disability Index

### Facet joint violation

Hereafter, we focus on whether the extra-facet puncture procedure could diminish the incidence of facet joint violation. As expected, a total of 26 facet joints (21.14%) were violated in traditional puncture group while there was no case with facet joint violation in extra-facet puncture group. As a whole, T9 (66.67%) is the most vulnerable vertebrae to being violated by the traditional puncture procedure followed by T11 (50%) and T12 (30%). The frequency of FJV was also common in the T10 vertebrae (28.57%). Overall, the rate of FJV in thoracic vertebrae (35.42%) is obviously higher than that in the lumbar vertebrae (12.0%). Besides, no facet joint was violated in L5 vertebrae (Table 3).

**Table 3:** Comparisons of postoperative parameters between traditional puncture PVP and extra-facet puncture PVP (*x* ± *s*, *n* = 242)

Parameter	t-PVP (*n* = 123)	ef-PVP (*n* = 119)	*p*
Bone cement volume (mL)	6.08 ± 1.44	5.95 ± 1.51	0.46^U^
Bone cement distribution			
Symmetrical	65 (52.85%)	90 (75.63%)	< 0.001^C^
Eccentric	42 (34.15%)	25 (21.01%)	
Unilateral	16 (13.01%)	4 (3.36%)	
Bone cement leakage	87 (70.73%)	84 (70.59%)	> 0.99^F^
Segmental kyphotic angle	9.85 ± 4.21	10.31 ± 4.93	0.43^T^
Vertebral compression ratio	25.63 ± 11.57	27.31 ± 11.81	0.32^U^
FJV	26 (21.14%)	0 (0%)	< 0.001^F^
T9	2 (66.67%)	0 (0%)	
T10	2 (28.57%)	0 (0%)	
T11	4 (50%)	0 (0%)	
T12	9 (30.0%)	0 (0%)	
L1	4 (10.81%)	0 (0%)	
L2	3 (12.5%)	0 (0%)	
L3	1 (14.28%)	0 (0%)	
L4	1 (16.67%)	0 (0%)	
L5	0 (0%)	0 (0%)	

*FJV* Facet joint violation

The symbol ‘U’ indicates the data was analyzed by the unpaired Mann–Whitney U test; The symbol ‘T’ indicates the data was analyzed by the unpaired t test; The symbol ‘F’ indicates the data was analyzed by the Fisher’s exact test; The symbol ‘C’ indicates the data was analyzed by the Chi-square test

*t-PVP* Traditional puncture percutaneous vertebroplasty, *ef-PVP* Extra-facet puncture percutaneous vertebroplasty

### Complications

**Fig. 2:**
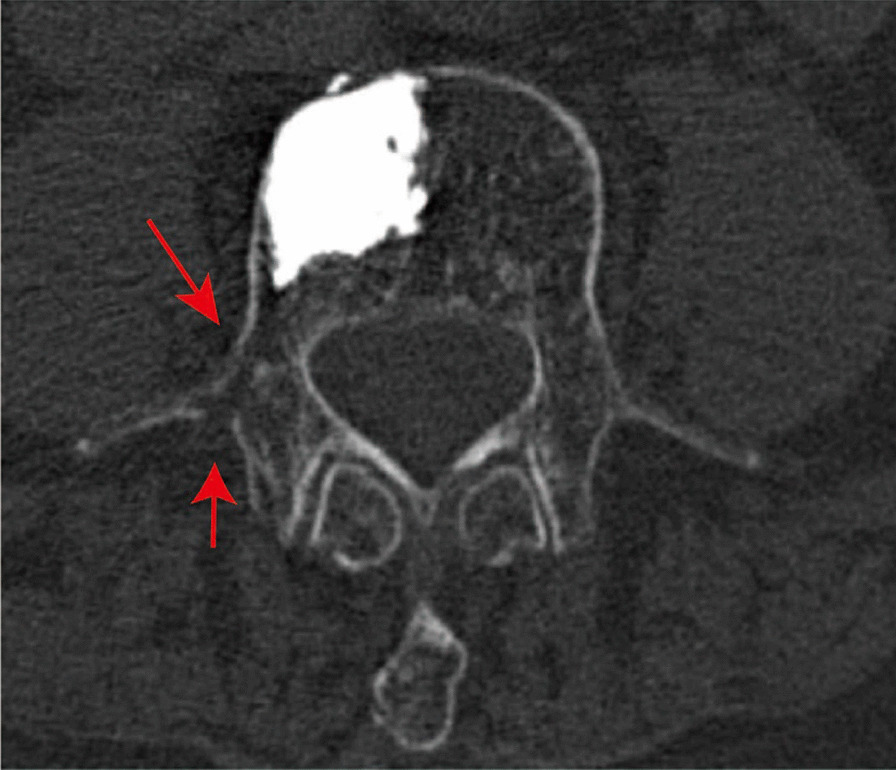
Extra-facet puncture PVP surgery with transverse process fracture. Female, 64 years old, who suffered L4 vertebral compression fracture and accepted extra-facet puncture PVP surgery. The postoperative CT transversal image showed L4 transverse process fracture

Cement leakage was observed in both traditional puncture group and extra-facet puncture group. There was no difference in the rates of cement leakage between the two groups (Table 3, *p* > 0.99). Pulmonary embolism, infection and bone cement allergy were not observed in this study. Accidentally, we found one case with transverse process fracture in extra-facet puncture group (Fig. 2).

### Bone cement distribution pattern

Notably, distribution and morphometric characteristics of bone cement on coronal planes are different in the two groups (Table 3, *p* < 0.001). In extra-facet puncture group, bone cement distribution was seen symmetrical in 90 patients (75.63%), eccentric in 25 patients (21.01%) and unilateral in 4 patients (3.36%). In traditional puncture group, symmetrical cement distribution was showed in 65 patients (52.85%), eccentric distribution in 42 patients (34.15%) and unilateral distribution in 16 patients (13.01%). Although symmetrical distribution was the most common pattern in traditional puncture group, eccentric and unilateral distribution are more prevalent in traditional puncture group compared to extra-facet puncture group.

### Comparison of FJV group and non-FJV group

**Fig. 3:**
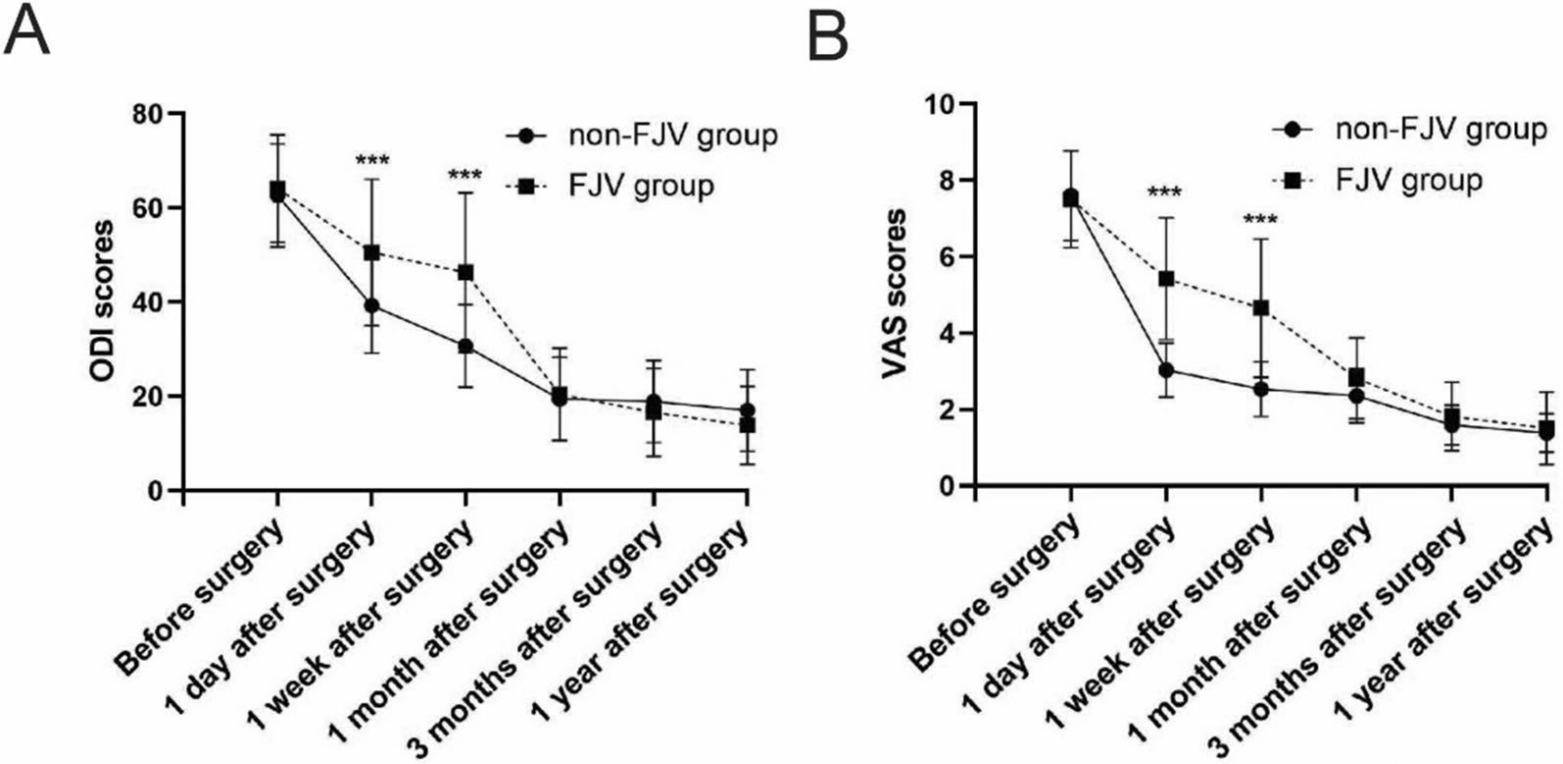
Comparison of the FJV group and the non-FJV group according to the ODI (**A**) and VAS (**B**) scores

Patients were regrouped according to the presence of facet joint violation. Of postoperative VAS and ODI scores, both groups showed significant decreases (Table 4). However, the VAS and ODI scores in the FJV group were significantly higher than those in the non-FJV group 1 day after surgery (*p* < 0.05). Within a month’s follow-up, the FJV group also had significantly higher scores in the VAS and ODI than the non-FJV group. Furthermore, differences of VAS and ODI scores between the two groups were not statistically significant from postoperative 1–12 months (*p* > 0.05) (Fig. 3).

**Table 4:** Comparisons of postoperative VAS and ODI scores in the FJV-group and the non-FJV group (*x* ± *s*, *n* = 217)

	VAS			ODI		
	FJV (*n* = 26)	Non-FJV (*n* = 191)	*p*	FJV (*n* = 26)	Non-FJV (*n* = 191)	*p*
Before surgery	7.50 ± 1.27	7.60 ± 1.18	0.63^U^	64.08 ± 11.40	62.65 ± 10.97	0.518^U^
1 day after surgery	5.42 ± 1.60	3.03 ± 0.71	< 0.001^U^	50.58 ± 15.57	39.28 ± 10.12	< 0.001^T^
1 week after surgery	4.65 ± 1.81	2.53 ± 0.72	< 0.001^U^	46.27 ± 16.96	30.71 ± 8.76	< 0.001^T^
1 month after surgery	2.81 ± 1.06	2.35 ± 0.71	0.063^U^	20.46 ± 9.82	19.43 ± 8.84	0.582^T^
3 months after surgery	1.81 ± 0.90	1.59 ± 0.52	0.260^U^	16.54 ± 9.33	18.93 ± 8.77	0.197^T^
1 year after surgery	1.50 ± 0.95	1.38 ± 0.50	0.359^U^	13.85 ± 8.31	17.04 ± 8.66	0.078^T^

The symbol ‘U’ indicates the data was analyzed by the unpaired Mann–Whitney U test; The symbol ‘T’ indicates the data was analyzed by the unpaired t test

*FJV* Facet joint violation

### Clinical outcomes

The clinical outcomes were assessed by VAS and ODI scores. As well, the MCID was used for evaluating the proportion of patients achieving significant pain relief in the two groups. No significant difference in VAS and ODI score was observed in either group preoperatively. Pain was clearly relieved after PVP surgery in both groups. Both VAS and ODI scores dropped with longer follow-up time in the two groups. Also, no significant difference was observed in the two groups from postoperative 1 day to 1 year after surgery regarding to the VAS scores (*p* > 0.05). The ODI scores in extra-facet puncture group were lower compared to traditional puncture group 1 month after surgery (Table 5, *p* = 0.015).

**Table 5:** Comparisons of postoperative VAS and ODI scores between traditional puncture PVP and extra-facet puncture PVP (*x* ± *s*, *n* = 217)

	VAS			ODI		
	t-PVP (*n* = 112)	ef-PVP (*n* = 105)	*p*	t-PVP (*n* = 112)	ef-PVP (*n* = 105)	*p*
1 day after surgery	3.50 ± 1.44	3.12 ± 0.72	0.294^U^	41.38 ± 12.54	39.84 ± 10.22	0.323^T^
1 week after surgery	2.98 ± 1.44	2.57 ± 0.66	0.053^U^	33.46 ± 13.05	31.64 ± 8.89	0.629^U^
1 month after surgery	2.43 ± 0.85	2.37 ± 0.68	0.693^U^	20.98 ± 8.76	18.03 ± 8.92	0.015^T^
3 months after surgery	1.60 ± 0.62	1.64 ± 0.54	0.468^U^	19.68 ± 8.81	17.54 ± 8.81	0.076^T^
1 year after surgery	1.33 ± 0.61	1.46 ± 0.52	0.069^U^	17.61 ± 8.61	15.64 ± 8.65	0.095^T^

The symbol ‘U’ indicates the data was analyzed by the unpaired Mann–Whitney U test; The symbol ‘T’ indicates the data was analyzed by the unpaired t test

The symbol ‘F’ indicates the data was analyzed by the Fisher’s exact test

*''t-PVP* Traditional puncture percutaneous vertebroplasty, *ef-PVP* Extra-facet puncture percutaneous vertebroplasty

Furthermore, the proportion of patients with immediate postoperative pain relief was significantly higher in extra-facet puncture as assessed by MCID of VAS (*n* = 94, 89.52%) (Table 6, *p* = 0.003). This percentage decreased to 73.21% (82 patients) in traditional puncture group. However, 1 month after surgery, differences were no longer detected between groups according to MCID of VAS and ODI scores.

**Table 6:** Comparisons of the proportion of patients achieving MCID in VAS and ODI scores between traditional puncture PVP and extra-facet puncture PVP (*n* = 217)

	VAS			ODI		
	t-PVP (*n* = 112)	ef-PVP (*n* = 105)	*p*	t-PVP (*n* = 112)	ef-PVP (*n* = 105)	*p*
1 day after surgery	82 (73.21%)	94 (89.52%)	0.003^F^	89 (79.46%)	98 (93.33%)	0.003^F^
1 week after surgery	87 (77.68%)	96 (91.43%)	0.008^F^	101 (90.18%)	104 (99.05%)	0.005^F^
1 month after surgery	101 (90.18%)	100 (95.24%)	0.197^F^	112 (100%)	105 (100%)	–
3 months after surgery	111 (99.11%)	105 (100%)	> 0.999^F^	112 (100%)	105 (100%)	–
1 year after surgery	111 (99.11%)	105 (100%)	> 0.999^F^	112 (100%)	105 (100%)	–

The symbol ‘U’ indicates the data was analyzed by the unpaired Mann–Whitney U test; The symbol ‘T’ indicates the data was analyzed by the unpaired t test

The symbol ‘F’ indicates the data was analyzed by the Fisher’s exact test

*MCID* minimal clinically important difference, *t-PVP* Traditional puncture percutaneous vertebroplasty, *ef-PVP* Extra-facet puncture percutaneous vertebroplasty

### Factors in predicting clinical outcomes between the two groups

To define factors influencing clinical efficacy between the two groups, we next performed an analysis after filtering patients with some potential known risk factors for postoperative residual back such as intravertebral vacuum cleft, posterior fascia oedema, multiple segment PVP and overly low cement volumes. There was a significant difference in the incidence of FJV and bone cement distribution between groups, both factors were analyzed and determined which was the more responsible predictor of residual back pain in patients underwent traditional puncture or extra-facet puncture PVP surgery.

Condition screening was performed and 81 (72.3%) patients in extra-facet puncture group and 79 (75.2%) patients in traditional puncture group were remained. As a result, although both factors affected the clinical outcome, FJV decreased treatment satisfaction at stronger levels of significance (OR = 16.38, *p* < 0.001) as proved by multivariable logistic regression (Table 7).

**Table 7:** Odds ratio (OR), 95% CI, *P* Value association using multivariable logistic regression models for residual back pain after PVP surgery including FJV and bone cement distribution pattern (*x* ± *s*, *n* = 160)

Parameter	*P*	OR	95% CI
FJV	< 0.001	16.38	5.144–52.167
Symmetrical	0.062		
Eccentric	0.773	1.156	0.432–3.094
Unilateral	0.020	5.576	1.318–23.583

*FJV* Facet joint violation

## Discussion

OVCFs have triggered significant societal issues and require proactive osteoporosis treatment [21, 22]. Over the last few decades, PVP has emerged as one of the fastest-evolving techniques in spine surgery [23]. Characterized by minimally invasive and immediate pain relief, PVP is the most widely used treatment for osteoporotic vertebral compression fractures [24, 25]. In spite of this, there are still some procedural complications such as cemented vertebral recollapse, adjacent vertebral fractures, cement leakage as well as facet joint violation [26–31]. Furthermore, some studied reported that unsatisfied bone cement distribution, low bone cement volume and individual factors such as fascia oedema, paraspinal muscle degeneration and intervertebral cleft could also cause residual back pain and adversely affected the patient's quality of life [12].

FJVs have a reported incidence of 15.9% and cause significant residual pain after PVP surgery. However, how to avoid or decrease the occurrence of FJV has not been mentioned [31]. In this study, we proposed and employed an extra-facet trajectory to protect the facet joint from violating. The trajectory between the lateral margin of facet joints and the lateral wall of pedicle was designed preoperatively according to CT scan. Moreover, the distance from the entry point to the lateral facet border was measured according to preoperative CT scan.

As a result, extra-facet puncture significantly decreased the incidence of FJV as shown in Table 3. Furthermore, it was demonstrated that VAS and ODI scores in the non-FJV group were significantly lower than those in the FJV group. However, we noted that though differences in VAS and ODI scores were observed between traditional puncture group and extra-facet puncture group, the improvements in extra-facet puncture group were not impressive and significant. Major, but not exclusive, the small proportion of FJV should be responsible for this. Hence, we introduced the MCID to assess the clinal outcomes in the two groups. Under the definition of great pain relief is achieving MCID in VAS scores, the proportion of patients with great pain relief in extra-facet puncture group was obvious higher than that in traditional puncture group (*p* = 0.003) at the first postoperative day (Table 6).

Although the results of extra-facet puncture were promising, generalization of the outcomes must be considered carefully. Moreover, the shortcomings of extra-facet puncture merits serious discussion. After reviewing the data of patients who did not get obvious pain relief after surgery in extra-facet puncture group, we found one patient suffered transverse process facture. Also, there are some risks of damage to lateral structures during extra-facet puncture.

Bone cement distribution is considered as an independent predictor for residual back pain in patients underwent PVP surgery. Li et.al reported that the blocky distribution of bone cement may increase the incidence of residual back pain after PVP surgery [12]. Moreover, Wang et.al showed that separated cement distribution was a strong risk factor in predicting residual back pain [31]. We divided the bone cement distribution into three types: symmetrical distribution, eccentric distribution and unilateral distribution. Unilateral distribution may cause subsequent residual pain. Extra-facet puncture can significantly increase the probability of cement symmetric distribution.

**Fig. 4:**
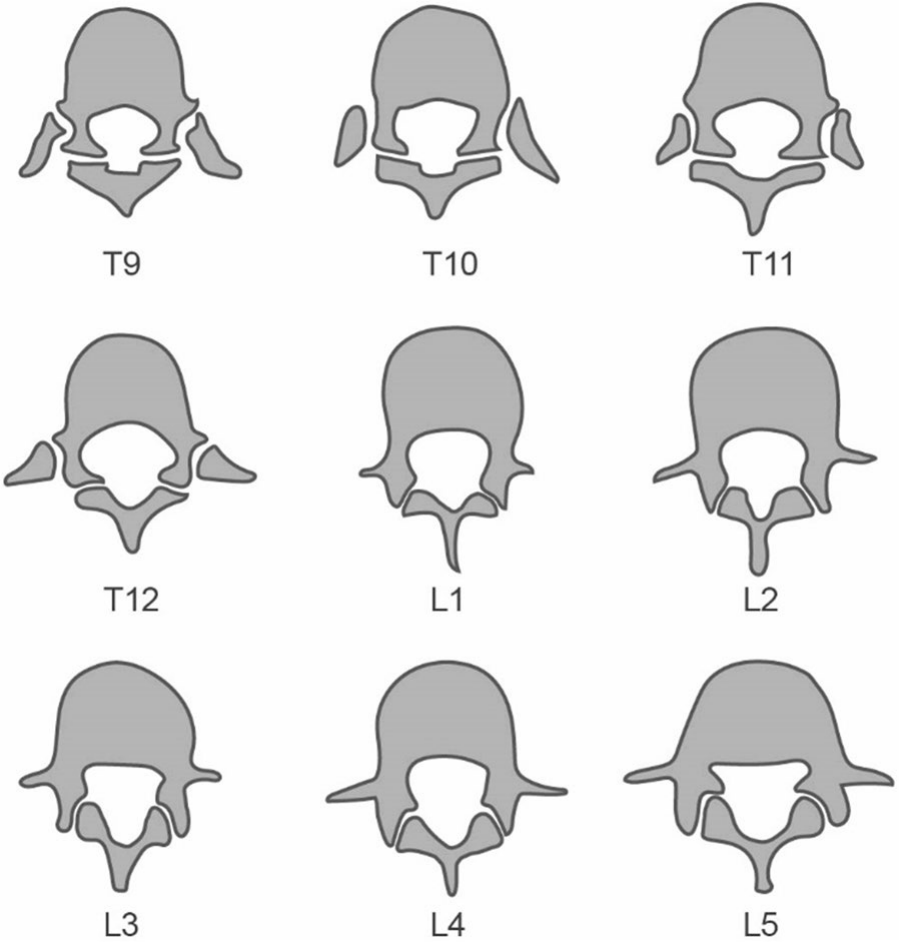
A schematic representation of the orientation of facet joints from T9 to L5

**Fig. 5:**
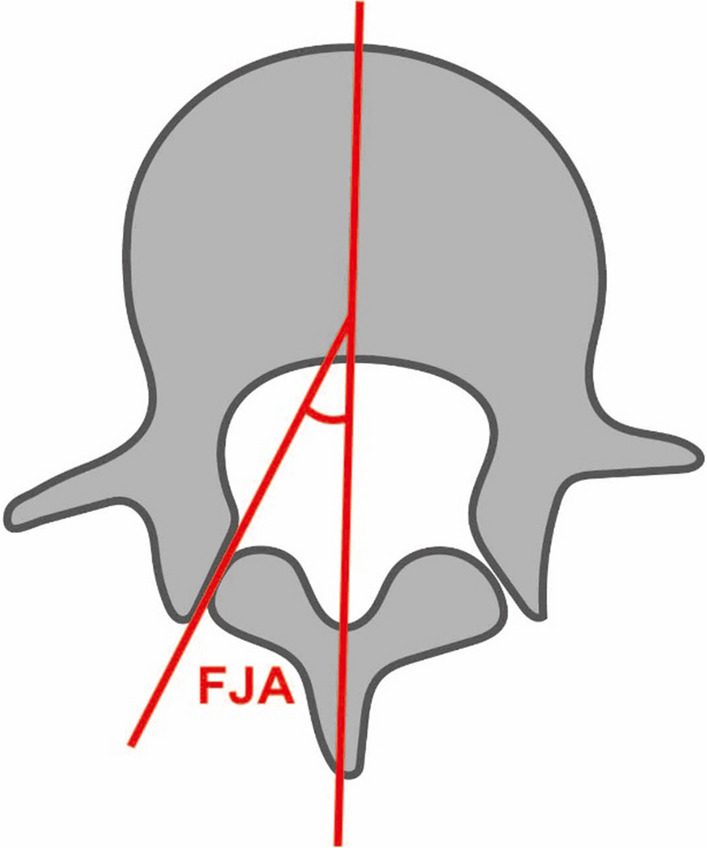
A schematic representation of the measure of FJA

Apart from that, we studied the orientation of facet joints at thoracolumbar segments aiming to unmask the connection between FJV and the morphology of the thoracolumbar facet joints. As a result of the change in direction of facet joints at the thoracolumbar junction, the orientation of the facet joints changes from the sagittal plane to the coronal plane abruptly (Fig. 4). This also leads to a sudden increase of the facet joint angle (FJA) in thoracic vertebrae (Fig. 5). A high-FJA is an independent risk factor of FJV [32]. Taking this into account, the conclusion can be drawn that traditional puncture in thoracic vertebrae is more prone to violate the facet joint. At the same time, our findings were consistent with this speculation shown in Table 3.

## Limitations

There is no patient with upper thoracic spine fracture and whether the extra-facet puncture PVP is suitable for the treatment of upper thoracic spine fracture remains unknown. Moreover, the incidence rates of FJV were not high so the sample size in the FJV group was small.

## Conclusions

Extra-facet puncture can decrease the risk of FJV and it also has the advantage of more satisfied bone cement distribution. The large sample, multi-center, randomized controlled trial is needed to confirm the efficacy of extra-facet puncture during PVP in the treatment of OVCFs.

## Data Availability

Data is available from the corresponding author.
